# Surveillance of Parrot Bornavirus in Taiwan Captive *Psittaciformes*

**DOI:** 10.3390/v16050805

**Published:** 2024-05-18

**Authors:** Brian Harvey Avanceña Villanueva, Jin-Yang Chen, Pei-Ju Lin, Hoang Minh, Van Phan Le, Yu-Chang Tyan, Jen-Pin Chuang, Kuo-Pin Chuang

**Affiliations:** 1 International Degree Program in Animal Vaccine Technology, International College, National Pingtung University of Science and Technology, Pingtung 912, Taiwan; j11285355@mail.npust.edu.tw; 2 Graduate Institute of Animal Vaccine Technology, College of Veterinary Medicine, National Pingtung University of Science and Technology, Pingtung 912, Taiwan; m11124010@mail.npust.edu.tw; 3 Livestock Disease Control Center of Chiayi County, Chiayi 612, Taiwan; a0934030008@gmail.com; 4 Department of Veterinary Medicine, National Chiayi University, Chiayi 600, Taiwan; 5 Department of Anatomy and Histology, Faculty of Veterinary Medicine, Vietnam National University of Agriculture, Hanoi 100000, Vietnam; hoangminh@vnua.edu.vn; 6 Department of Microbiology and Infectious Diseases, Faculty of Veterinary Medicine, Vietnam National University of Agriculture, Hanoi 100000, Vietnam; letranphan@vnua.edu.vn; 7 Department of Medical Imaging and Radiological Sciences, Kaohsiung Medical University, Kaohsiung 807, Taiwan; 8 Department of Medical Research, Kaohsiung Medical University Hospital, Kaohsiung 807, Taiwan; 9 Center for Cancer Research, Kaohsiung Medical University, Kaohsiung 807, Taiwan; 10 Center for Tropical Medicine and Infectious Disease Research, Kaohsiung Medical University, Kaohsiung 807, Taiwan; 11 Chiayi Hospital, Ministry of Health and Welfare, Chiayi 600, Taiwan; 12 Department of Surgery, Faculty of Medicine, College of Medicine, National Cheng Kung University, Tainan 701, Taiwan; 13 Department of Surgery, National Cheng Kung University Hospital, Tainan 704, Taiwan; 14 School of Medicine, Kaohsiung Medical University, Kaohsiung 807, Taiwan; 15 School of Dentistry, Kaohsiung Medical University, Kaohsiung 807, Taiwan; 16 Companion Animal Research Center, National Pingtung University of Science and Technology, Pingtung 912, Taiwan

**Keywords:** parrot bornavirus, proventricular dilatation diseases, prevalence rate, infectious disease

## Abstract

Parrot bornavirus (PaBV) is an infectious disease linked with proventricular dilatation disease (PDD) with severe digestive and neurological symptoms affecting psittacine birds. Despite its detection in 2008, PaBV prevalence in Taiwan remains unexplored. Taiwan is one of the leading psittacine bird breeders; hence, understanding the distribution of PaBV aids preventive measures in controlling spread, early disease recognition, epidemiology, and transmission dynamics. Here, we aimed to detect the prevalence rate of PaBV and assess its genetic variation in Taiwan. Among 124 psittacine birds tested, fifty-seven were PaBV-positive, a prevalence rate of 45.97%. Most of the PaBV infections were adult psittacine birds, with five birds surviving the infection, resulting in a low survival rate (8.77%). A year of parrot bornavirus surveillance presented a seasonal pattern, with peak PaBV infection rates occurring in the spring season (68%) and the least in the summer season (25%), indicating the occurrence of PaBV infections linked to seasonal factors. Histopathology reveals severe meningoencephalitis in the cerebellum and dilated cardiomyopathy of the heart in psittacine birds who suffered from PDD. Three brain samples underwent X/P gene sequencing, revealing PaBV-2 and PaBV-4 viral genotypes through phylogenetic analyses. This underscores the necessity for ongoing PaBV surveillance and further investigation into its pathophysiology and transmission routes.

## 1. Introduction

Parrot bornaviruses (PaBV), namely parrot bornaviruses 1 to 8, are from the family of *Bornaviridae,* and the genus of *Orthobornavirus* has two viral species: *Orthobornavirus alphapsittaciforme* and *Orthobornavirus betapsittaciforme* [1]. *Orthobornavirus alphapsittaciforme* has six genotypes, which are PaBV-1 to -4, -7, and -8, while *Orthobornavirus betapsittaciforme* has two genotypes, PaBV-5 and -6 [1]. Although PaBV-1 to -8 are known in psittacine/parrot birds (order Psittaciformes), other avian bornaviruses host specific orders of birds. Canary bornaviruses 1 to 3 (CnBV-1 to CnBV-3) and munia bornavirus 1 (MuBV-1) belong to *Orthobornavirus serini* and estrildid finch bornavirus 1 (EsBV-1) belongs to *Orthobornavirus estrildidae* for passerine birds (order Passeriformes). In aquatic birds (orders Anseriformes and Charadriiformes), aquatic bornaviruses 1 and 2 (ABBV-1 and ABBV-2) are viral species of *Orthobornavirus avisaquaticae* [1]. Aside from avian host species, orthobornaviruses of mammalian and reptilian species were also reported. Orthobornaviruses of mammals such as Borna disease viruses 1 and 2 (BoDV-1 and -2) are viral species of *Orthobornavirus bornaense,* and variegated squirrel bornavirus 1 (VSBV-1) is a viral species of *Orthobornavirus sciuri*. Lastly, orthobornaviruses in snakes such as Caribbean watersnake bornavirus (CWBV) and Mexican black-tailed rattlesnake bornavirus (MRBV) are viral species of *Orthobornavirus caenophidiae* and Loveridge’s garter snake virus 1 (LGSV-1) is a viral species of *Orthobornavirus elapsoideae* [1]. Recently, a new avian bornavirus was reported, barn owl bornavirus 1 (BoBV-1), with 83% viral sequence similarity with CnBV-2 and being detected in a barn owl (*Tyto* alba), suggesting a wider host range of *Orthobornavirus serini* [2]. Another report detected that avian bornaviruses in rgw Himalayan monal (*Lophophorus impedance*) and white-bellied caique (*Pionites leucogaster*) housed outside with eight other parrots (*Amazona* spp. and *Cacatua* spp.) have 100% matrix gene similarity with PaBV-4 [3]. Further surveillance, prevalence, and sequence reports on avian bornavirus are beneficial in further classifying and understanding their host range.

Parrot bornavirus is the causative agent of proventricular dilatation disease (PDD), and infection leads to a chronic neurological and digestive disorder in psittacine birds. PaBV presence has been reported in Brazil [4,5,6]; Germany [7,8,9,10,11]; France and Spain [9]; Portugal [12]; the Netherlands [8]; Sweden [13]; Austria, Switzerland, Hungary, and Australia [14]; the United States [15,16,17,18]; Canada [19,20]; South Africa [21]; the Czech Republic and Slovakia [22]; Qatar [23]; Israel [18]; Malaysia [24]; Thailand [25]; China [26]; South Korea [27]; and Japan [28,29]. The detection rates of PaBV vary significantly among these countries. Viral RNA detection through RT-qPCR has become the standard due to its low contamination risk and high sensitivity and specificity, while feces samples have been the most available for PaBV detection [1,30]. Currently, no studies have been available regarding the detection of PaBV infection cases in Taiwan.

In Taiwan, psittacine birds are the main pet birds and the majority of psittacine bird owners have more than one psittacine bird. Taiwan is also one of the leading exporters of parrots in global trade and has the largest number of naturalized or breeding psittacine birds [31,32]. With the great population of psittacine birds and the global lead in breeding, it is essential to understand the presence of infectious-disease-causing agents such as PaBV. This will provide insight into initial evidence of the potential risk that PaBV carries, especially in Taiwan’s psittacine birds.

In Taiwan, there has been scarce available information regarding PaBV infection cases. Moreover, due to the high economic value of psittacine birds which are favored as companion pets in Taiwan, it is essential to further investigate the infectious virus, thus highlighting the importance of PaBV investigation. Hence, the presence and surveillance of PaBV across Taiwan are of significant importance in PaBV investigations and prevention. In this study, we screened one hundred twenty-four feces of captive psittacine birds collected for a year to report the prevalence rate of PaBV infection cases in Taiwan. Likewise, the sequencing of the PaBV X/P gene from a brain sample revealed the genotypes present in Taiwan. Furthermore, histopathology and radiograph images provide some insight into the nature of the proventriculus, cerebellum, and heart due to PaBV infection. This study underscores the critical need for ongoing surveillance and investigation into PaBV infection cases in Taiwan, particularly given the economic significance and popularity of psittacine birds as companion pets. By understanding the prevalence rates, genotypes, and pathological effects of PaBV, we can better inform prevention strategies and safeguard both psittacine birds of Taiwan.

## 2. Materials and Methods

### 2.1. Sample Collection

One hundred twenty-four psittacine feces samples used in this study were freshly collected by the veterinarian at Xing Yu Animal Hospital, Linyuan District, Kaohsiung City, Taiwan, or directly from the private psittacine owner’s or breeder’s house where the psittacine bird is housed and following the permission of the psittacine owners. For psittacine birds housed together, they were separated first and fresh feces were collected immediately after dropping. Clinal organs such as the brain, lung, pancreas, stomach, intestine, liver, eye, kidney, heart, muscle, and proventriculus from three psittacine birds that suffered from PaBV were donated for necropsy and RT-qPCR detection in different organ tissue. All the samples were placed in an ice bath right after collection and stored in a −80 °C Ultra-Low-Temperature Freezer (MDF-DU302VX-PA, TwinGuard, PHCBI, Gunma, Japan). The collection and sample handling guidelines were followed and approved by NPUST-IACUC. The sample collection started in June of 2022 and lasted until May 2023. Several psittacine birds with severe clinical symptoms of proventricular dilation disease (PDD) were screened through X-ray scans (E7239X, Rotanode^TM^, Toshiba, Otawara, Japan) for their proventriculus size. Feces or clinical organ samples were prepared as 20% homogenates with 1x Phosphate-Buffered Solution (PBS), incubated for 30 min, and centrifuged (Centrisart A-14C, Sartorius, Göttingen, Germany) for 12,000× *g* for 15 min at 4 °C, and then the clarified supernatants were collected for RNA extraction and the excess was stored at −80 °C.

The 124 collected feces samples were as follows: 2 were from Taipei City (2/124; 1.61%), 2 were from Taoyuan City (2/124; 1.61%), 9 were from Taichung City (9/124; 7.26%), 1 was from Changhua County (1/124; 0.81%), 3 were from Yunlin County (3/124; 2.42%), 1 was from Nantou County (1/124; 0.81%), 7 were from Chiayi City (7/124; 5.65%), 3 was from Chiayi County (3/124; 2.42%), 16 were from Tainan City (16/124; 12.9%), 48 were from Kaohsiung City (48/124; 38.71%), and 32 were from Pingtung County (32/124; 25.81%). Altogether, the species of psittacine birds sampled were as follows: 2 *Agapornis* sp. (2/124; 1.61%), 1 *Agapornis pullarius* (1/124; 0.81%), 2 *Amazona aestiva* (2/124; 1.61%), 9 *Amazona ochrocephala* (9/124; 7.26%), 2 *Ara severus* (2/124; 1.61%), 3 *Ara auricollis* (3/124; 2.42%), 2 *Cacatua alba* (2/124; 1.61%), 3 *Cacatua ducorpsii* (3/124; 2.42%), 3 *Cacatua ophthalmica* (3/124; 2.42%), 1 *Cacatua moluccensis* (1/124; 0.81%), 10 *Diopsittaca nobilis* (10/124; 8.06%), 11 *Aratinga solstitialis* (11/124; 8.87%), 7 *Eclectus roratus* (7/124; 5.65%), 1 *Eolophus roseicapillus* (1/124; 0.81%), 1 *Ara ararauna* (1/124; 0.81%), 4 *Melopsittacus undulatus* (4/124; 3.23%), 7 *Myiopsitta monachus* (7/124; 5.65%), 16 *Nymphicus hollandicus* (16/124; 12.9%), 4 *Pionites leucogaster* (4/124; 3.23%), 2 *Pionus maximiliani* (2/124; 1.61%), 1 *Poicephalus gulielmi* (1/124; 0.81%), 5 *Psittacula krameri* (5/124; 4.03%), 26 *Psittacus erithacus* (26/124; 20.97%), and 1 *Pyrrhura molinae* (1/124; 0.81%).

### 2.2. Detection of Parrot Bornavirus

#### 2.2.1. RNA Extraction

The RNA was extracted from the supernatant samples of 1XPBS homogenized feces of 124 psittacine birds following the manufacturer’s instructions of R.T.U REzol^TM^ C&T (Protech, Taipei, Taiwan, KP200CT) with slight modification. An amount of 500 μL of the feces supernatant was mixed with 500 μL of REzol^TM^ C&T in 1.5 mL sterile microcentrifuge tubes (JetBiofil, Guangzhou, China, CFT100015), shaken vigorously for 15 s, and incubated for 5 min, and then 100 μL of chloroform was added, shaken for 15 s, incubated for 2 min, and centrifuged for 12,000× *g* for 15 min at 4 °C. A 500 μL colorless upper aqueous phase was collected and transferred in a new microcentrifuge tube, added with 500 μL of isopropanol (Honeywell Burdick & Jackson, Ulsan, Republic of Korea, AH323-4) with gentle mixing, incubated for 10 min, and centrifuged for 12, 000× *g* for 10 min at 4 °C, and isopropanol supernatant was discarded. The RNA precipitate pellets were washed with 1 mL of 75% ethanol (Echo, Miaoli, Taiwan) by vortex-mixing for 2 s and centrifuged for 5 min at 12,000× *g* for 4 °C, ethanol supernatant was discarded, and RNA pellets were air-dried for 15 min or more if required. Dried RNA pellets were dissolved in 50 µL of UltraPure DEPC (Protech, Taipei, Taiwan, PT-P560-500) and quantified using NanoDrop2000 Spectrophotometer (Thermo Fisher Scientific, Wilmington, DE, USA).

#### 2.2.2. Reverse Transcription–Quantitative Polymerase Chain Reaction

The 124 freshly collected feces samples were extracted for RNA immediately and tested for PaBV through RT-qPCR. Clinical organ samples such as the brain, kidney, eyes, stomach, lung, intestine, liver, pancreas, and heart from three psittacine birds donated to our lab were also subjected to RT-qPCR. The RT-qPCR detection for parrot bornavirus (PaBV) was performed in a StepOne™ Real-Time PCR System Thermal Cycling Block (Applied Biosystems, Thermo Fisher Scientific, Marsiling, Singapore) using the qPCRBIO Probe 1-Step Go Hi-ROX kit (PCR Biosystems, London, UK, PB25.42) following the manufacturer’s instructions with slight modification. Each reaction contained 5 µL of 2x qPCRBIO Probe 1-Step Go Mix, 0.4 µL of reverse and forward primers each with concentrations of 10 µM, 0.2 µL of the probe at 10 µM concentration, 1.5 µL of UltraPure DEPC water (Protech, Taipei, Taiwan, PT-P560-500), and 2.5 µL of RNA extracted from feces samples in a total reaction volume of 10 µL. The thermal cycle was as follows: 45 °C for 10 min for reverse transcription, 95 °C for 2 min for polymerase activation, and 40 cycles of 95 °C for 5 s and 60 °C for 30 s for denaturation and annealing/extension. The primers and the probe (Genomics BioSci & Tech, New Taipei City, Taiwan) used for RT-qPCR detection were as follows: for the forward primer, BornaPCA3 (5′-GATCCGCAGACAGYACGT-3′) was used; for the reverse primer; BornaPCA6 (5′-GAGATCATGGANGGRTTCTT-3′) was used; and for the probe, BornaP_Fam (5′-FAM-CGAATWCCCAGGGAGGCYCT-BHQ1-3′) was used, specific for PaBV-1, PaBV-2, PaBV-4, and PaBV-7 detection with a product size of 125 bp [30].

#### 2.2.3. Gel Electrophoresis

The RT-qPCR products underwent gel electrophoresis to further confirm the detection of parrot bornavirus. An amount of 1.5 g of Biotechnology Grade Agarose I (VWR Chemicals, Solon, OH, USA, 97062-250) was dissolved in 100 mL of 0.5x Tris-borate-EDTA (TBE) solution, microwave-heated (RE-0711, Sampo, Taoyuan, Taiwan) for 2 min, shaken for 10 s, microwaved-heated for 2 min, shaken for 10 s, added with 5 µL of EtB”Out” Nucleic Acid Staining Solution 2.0 (Yeastern Biotech, Taipei City, Taiwan, FYD007-200P), shaken for 10 s to mix, and placed in a mold to solidify for 15 min. A Mupid^®^-2plus Submarine Electrophoresis System (Advance, Tokyo, Japan) was used for gel electrophoresis with a run time of 5 min for half voltage followed by 25 min for full voltage with 0.5x TBE running buffer, enough to submerge and cover the gel. The RT-qPCR products were added with 1 µL of 6x DNA Loading Dye (GeneMarkbiolab, Taichung, Taiwan, DL02), and 100 bp of DNA Ladder (Bio-Van, New Taipei City, Taiwan, M0100) was used. The gel band was viewed through a UV Transilluminator (MUV21-312, Major Science, Taoyuan City, Taiwan) compound with UV Imager (CI-01, Major Science, Taoyuan City, Taiwan), visualized with Major Science 1D Analysis software version 1.0 at 24x integration, and the gel band image was captured using a mono camera (MTV-12V6HE-R, Mintron, New Taipei City, Taiwan) with an 8.5–51 mm F1.2 lens (SSL85051M, Avenir Lens, Seiko, Tokyo, Japan).

### 2.3. Histopathological Examination

Donated clinical cerebellum and heart samples of deceased psittacine birds due to proventriculus dilatation disease were fixed with 10% phosphate buffer formalin solution and embedded in paraffin. A hematoxylin–eosin (H&E) stain was used for every 5 µm thick tissue sample. The samples were observed under a light microscope (Eclipse E200, Nikon, Shonan, Japan), and images were obtained using a Nikon DS-L2 camera (Nikon, Shonan, Japan).

### 2.4. Transmission Electron Microscope

1x PBS-homogenized brain samples were centrifuged for 10 min at 4000× *g* at 4 °C followed by 12,000× *g* at 4 °C for 30 min. The supernatant was passed through a 0.22 µm Millex^®^-GV filter (Merck Millipore, Carrigtwohill, Ireland, PR05099). Then, the virus was concentrated through polyethylene glycol 6000 (Alfa Aesar, Karlsruhe, Germany, 10212393) with 10% *w*/*v*, centrifuged at 3500× *g* for 5 min, and then 1.45 g of NaCl (Showa Chemical, Tokyo, Japan, KGE-343A) at 1 M was added for every 20 mL of virus supernatant. It was incubated overnight with shaking at 4 °C. Afterwards, it was centrifuged in 10,000× *g* for 30 min, the supernatant was discarded, and pellets were dissolved with 1x PBS. The virus solution was allowed to settle in a formvar/carbon 200-mesh copper grid (Ted Pella, Redding, CA, USA, 01800-F) for 30 s and coated with 2% aqueous uranyl acetate solution (Spi-Chem, Structure Probe, West Chester, PA, USA, 02624-AB) for 45 s. Excess stain was removed and the specimen was examined using an H-7500 transmission electron microscope (Hitachi, Tokyo, Japan).

### 2.5. PCR Amplification of the X/P Region and Sequencing

Following the manufacturer’s instruction, RNA-extracted residue from three PaBV-positive donated brain samples was converted to cDNA using the qScript^®^ cDNA Synthesis Kit (Quantabio, Hilden, Germany, 95047). PaBV X/P region primer (forward: 5′-CTCAATGGCACGGCCCTC-3′ and reverse: 5′-GGCCATCCAGGAACAATTACC-3′) was designed through NCBI Primer-BLAST using PaBV sequences with accession ID NC_039189.1, NC_028106.1, FJ620690.1, EU781967.1, NC_030688.1, JX065209.1, GU249596.2, and NC_030689.1. The P Fast-Pfu 2X PCR SuperMix (AllBio, Taichung, Taiwan, ABTGMBP03-100) produced X/P amplicons following the manufacturer’s instructions. Each reaction has 1 µL of forward and reverse primer at 10 µM concentrations, 25 µL of P Fast-Pfu 2X PCR SuperMix, 18 µL of UltraPure DEPC water (Protech, Taipei, Taiwan, PT-P560-500), and 5 µL of cDNA in a total reaction of 50 µL. A Blue-Ray Biotech Turbo Cycler (Blue-Ray Biotech, Taipei City, Taiwan) was used for the PCR with thermal cycling conditions of 94 °C for 5 min as the initial denaturation, followed by 40 cycles of 94 °C for 30 s, 60 °C for 30 s, 72 °C for 1 min, and a final extension at 72 °C for 10 min. The PCR amplicon product size was 682 pb, amplicons were run in 1.5% agarose gel electrophoresis, and the band was sliced with a 200 mg weight and then sent to Genomics BioSci & Tech, New Taipei City, Taiwan, for sequencing. X/P amplicons were sequenced through the Sanger sequencing approach (Genomics BioSci & Tech, New Taipei City, Taiwan).

### 2.6. Phylogenetic Analysis

Alignments of X/P gene sequences were performed using the MUSCLE algorithm at 1000 bootstrap replicates of MEGA 11.0.13 software. Using available *Orthobornavirus* sequences, phylogenetic analyses were performed through the maximum likelihood algorithm and GTR + I model at 1000 bootstrap replicates.

## 3. Results

**Figure 1:**
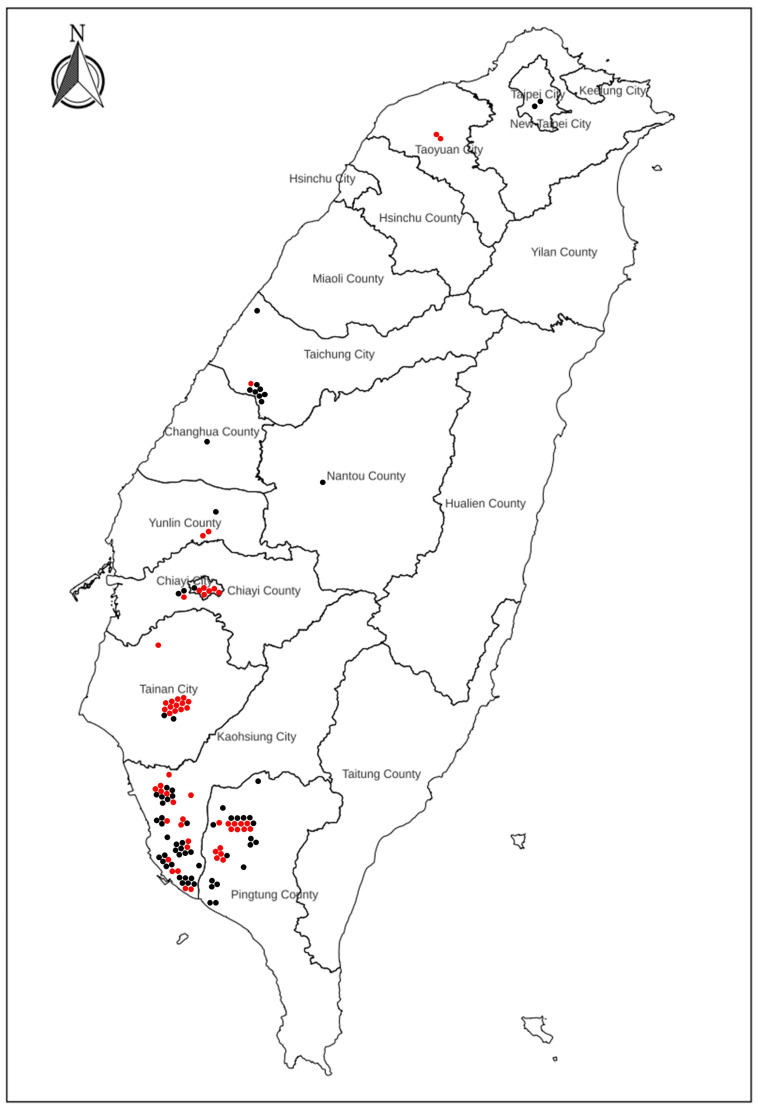
The map shows the geographical location of PaBV-positive (red dots) and PaBV-negative (black dots) psittacine birds in Taiwan; map was sourced from the National Land Surveying and Mapping Center (NLSC).

One hundred twenty-four (124) fecal samples were tested for PaBV, of which 2 were from Taipei City, 2 were from Taoyuan City, 9 were from Taichung City, 1 was from Changhua County, 3 were from Yunlin County, 1 was from Nantou County, 7 were from Chiayi City, 3 was from Chiayi County, 16 were from Tainan City, 48 were from Kaohsiung City, and 32 were from Pingtung County (Figure 1). Altogether, the feces-sampled psittacine birds were privately owned or breeding birds and were mostly contained in their appropriate cages. Not long after testing positive with PaBV RT-qPCR detection, the birds passed away (around 1 week–2 months). Most PaBV-positive birds were observed with digestive symptoms (loss of appetite, weight loss, emaciation, undigested seeds shedding in feces, diarrhea, proventriculus dilation, and delayed crop emptying) and neurological symptoms (incoordination, seizure, tremor, lameness, and retinitis).

**Figure 2:**
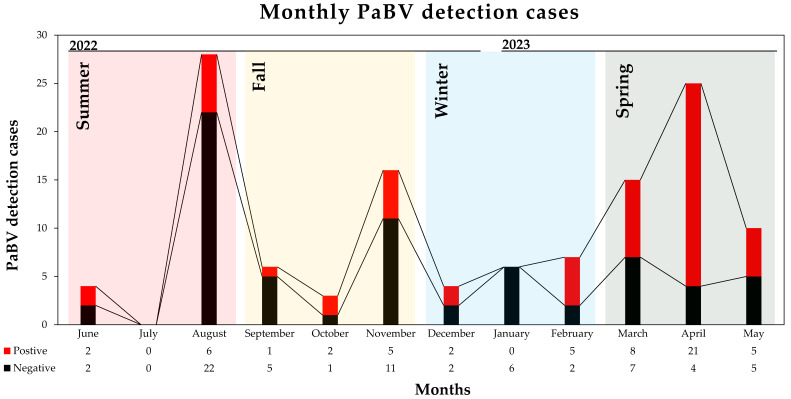
PaBV detection cases per month (June 2022 to May 2023); PaBV-positive case are in red while PaBV-negative cases are in black.

A total of 124 psittacine birds were subjected to PaBV detection, and 57 (57/124; 45.97%) were positive for PaBV. The C_T_ values ranged from 19.93 to 31.81. PaBV-positive cases were as follows: 2 from Taoyuan City (2/2; 100%), 1 from Taichung City (1/9; 11.11%), 2 from Yunlin County (2/3; 66.67%), 6 from Chiayi City (6/7; 85.71%), 1 from Chiayi County (1/1; 100%), 14 from Tainan City (14/16; 87.5%), 17 from Kaohsiung City (17/48; 35.42%), and 14 from Pingtung County (14/32; 43.75%) (Figure 1). Only five out of the fifty-seven PaBV-positive birds (5/57; 8.77%) survived PaBV infection, with observed proventricular dilatation, of which there were 2 from Tainan, 1 from Kaohsiung, and 2 from Pingtung, suggesting a high fatality rate (52/57; 91.23%) and mortality rate (52/124; 41.94%). Sample collection started from June of 2022 to May of 2023. The highest PaBV-positive rate was observed in April of 2023 (21/25; 84%) followed by October of 2022 and February of 2023 (2/3; 66.67% and 5/7; 71.4%), while zero positive cases were observed in January of 2023 (Figure 2). During the summer season (June to August), there were 25% (8/32) PaBV-positive cases, with 32% (8/25) during the fall season (September to November) and 41% (7/17) during the winter season (December to February), and an increasing trend was observed up to 68% (34/50) in the spring season (March to May). Most psittacine birds were companion birds, spending most of their time indoors or housed outdoors. These findings underscore the significant impact of PaBV infection on psittacine birds, highlighting the importance of continued monitoring and preventive measures to mitigate the high fatality and mortality rates associated with the disease.

PaBV-infection cases were found to be highest with *Psittacus erithacus* psittacine bird species (11/57; 19%) followed by *Nymphicus hollandicus* (8/57; 14%) and *Aratinga solstitialis* (8/57; 14%) (Table 1). The rest of the PaBV-positive rates per psittacine species were as follows: 7% (4/57) for each species of *Myiopsitta monachus*, *Diopsittaca nobilis*, and *Amazona ochrocephala*; 4% (2/57) for each species of *Cacatua ophthalmica*, *Eclectus roratus*, *Ara severus*, *Pionites leucogaster*, *Amazona aestiva*, *Agapornis* sp., and *Psittacula krameri*; and 2% (1/57) for each species of *Poicephalus gulielmi*, *Pyrrhura molinae*, *Melopsittacus undulatus*, and *Cacatua moluccensis*.

The ambiguity surrounding the age and sex of the psittacine birds often reflects on their adoption, mostly without prior information. Most of the PDD-suffering birds were adults, 1 year old and up. Based on thirty-one (31) identified sexes of the psittacine birds, 64.52% (20/31) were male and 35.48% (11/31) were female (Table 2). Mutual symptoms were weight loss, crop stasis, maldigestion, and proventriculus dilation followed by depression and regurgitation. Seizure was only observed in one identified male bird (case no. 2302013), while both ataxia and tremor were observed in 25% (5/20) of the males, and for females, and 18.18% (2/11) ataxia and 45.45% (5/11) tremor symptoms were observed.

**Figure 3:**
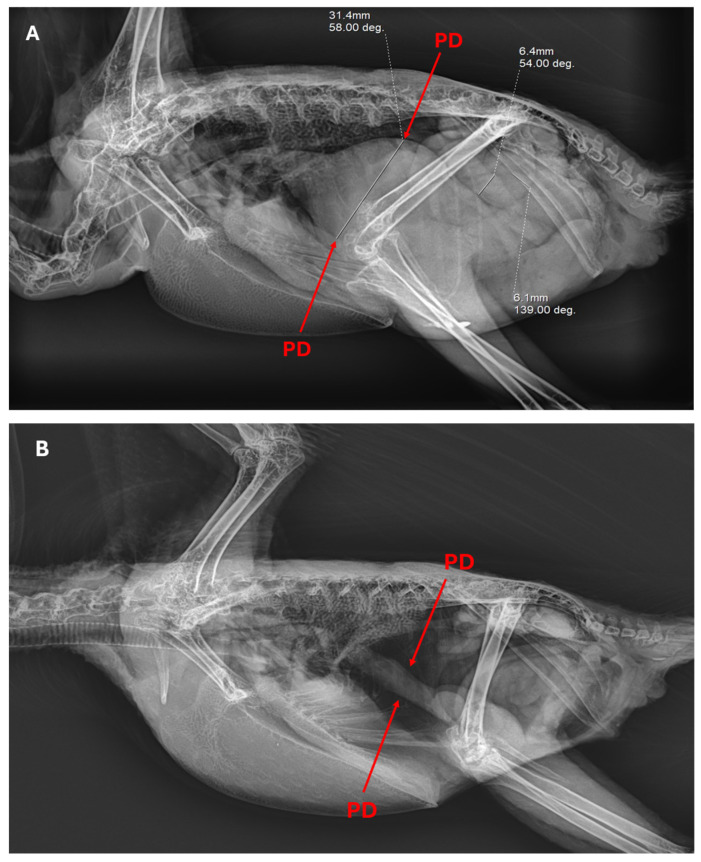
(**A**) X-ray scan of a proventriculus dilation (PD), 31.4 mm, and intestine, 6.4 mm and 6.1 mm; and (**B**) a normal proventriculus. Two radiographic images clearly illustrate the difference in the proventriculus diameter of a PDD-suffering psittacine bird compared to a healthy psittacine bird.

Additionally, psittacine birds with obvious and severe PDD clinical symptoms were subjected to X-ray scans to screen digestive organs, specifically proventriculus dilatation, observed in Figure 3A, while Figure 3B displays a proventriculus of a healthy psittacine bird. The proventriculus diameter could determine the short-term survival of psittacine birds with gastric implications. The normal range of the proventricular diameter-to-dorsoventral keel height ratio score was 0.200–0.476 [33,34], among the PDD-suffering birds a range from the proventricular diameter-to-dorsoventral keel height recorded exceeded the normal range, from 0.681–0.981 implying their poor survival.

**Figure 4:**
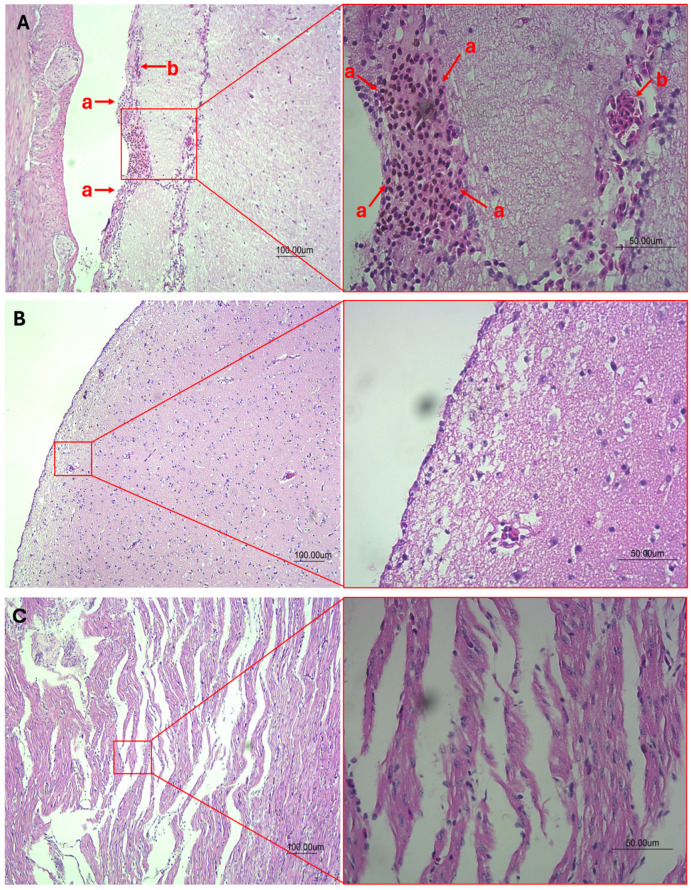
(**A**) Histopathological image at 10× and 40× magnification of the cerebellum with severe and diffuse lymphocytes indicating severe meningoencephalitis of PaBV-infected psittacine bird suffering from PDD/AGN (a: lymphocytes and b: red blood cells), and (**B**) histopathological image of the cerebellum of psittacine birds negative to PaBV infection or any signs of digestive or neurological disorders. (**C**) Histopathological image at 10× and 40× magnification of the heart from a PaBV-positive psittacine bird suffering from PDD/AGN with extensive degeneration to necrosis of the myocardium and dilated cardiomyopathy can be observed.

**Figure 5:**
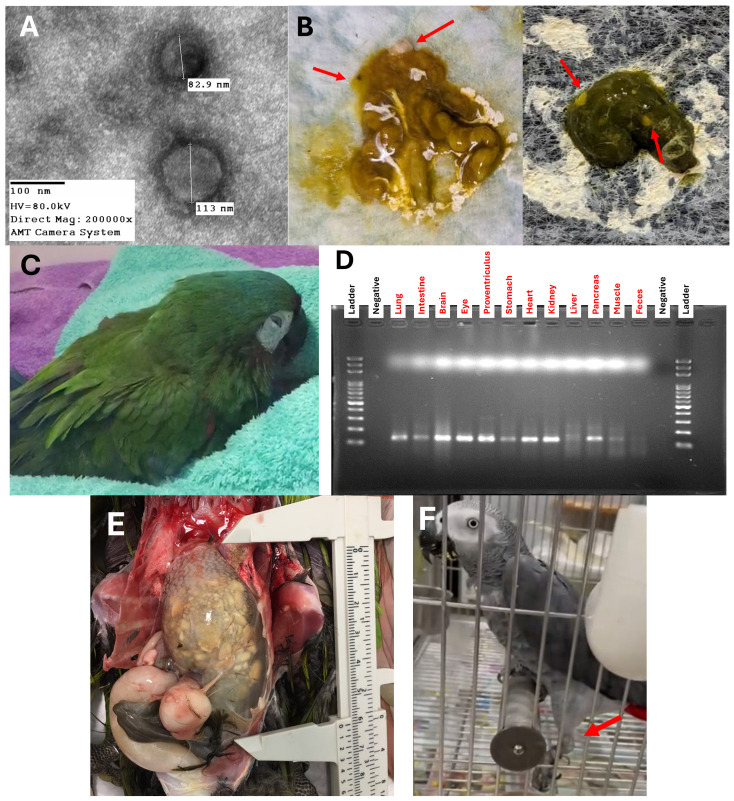
(**A**) Transmission electron microscopy image of the PaBV at 200,000× magnification, with a diameter of 82.9 nm and 113 nm. (**B**) Feces droppings with shedding of undigested seeds (red arrow). (**C**) Red-shouldered macaw (*Diopsittaca nobilis*) suffering from proventriculus dilation and showing neurological signs; discomfort can be seen in the twitching of the eyes. (**D**) Gel band image of the RT-qPCR product, 125 bp, for PaBV detection in lung, intestine, brain, eye, proventriculus, stomach, heart, kidney, liver, pancreas, muscle, and feces samples from case number 2301024. (**E**) Dilated proventriculus with a thin wall containing undigested seeds, about 6 cm in length, of a red-shouldered macaw (*Diopsittaca nobilis*) enduring proventriculus dilatation disease. (**F**) Photo of an African grey parrot (*Psittacus erithacus*) unable to balance right before it fell due to loss of control of the left leg (red arrow).

The elevated presence of lymphocytes in the cerebellum as shown in Figure 4A uncovers that PaBV-positive birds had a severe and diffused meningoencephalitis in contrast with a PaBV-negative cerebellum of a psittacine bird in Figure 4B. In Figure 4C, the psittacine bird suffered from PDD/AGN and PaBV-positive birds demonstrate dilated cardiomyopathy in the heart and extensive degeneration to necrosis of the myocardium. A transmission electron image of the PaBV is observed in Figure 5A, where the size ranges from 82.9 nm to 113 nm. Parrot bornaviruses are membrane viruses that allow them to enlarge or contract in size. The shedding of undigested seeds and diarrhea symptoms in feces were observed among PDD-suffering psittacine birds as a clinical symptom of gastric implications or digestive disorder (Figure 5B). At the same time, physiological symptoms such as tilting of the head, twitching of the eyes, and loss of balance were observed (Figure 5C,F). The twitching of the eyes and the rousing of the feathers indicate the discomfort of the bird (Figure 5C). Typical loss of balance is due to ataxia or loss of muscle, limiting the bird from grabbing and climbing. A 125 bp RT-qPCR product for PaBV detection was further confirmed in gel electrophoresis for gel band viewing. Figure 5D displays the gel band image for the lung, intestine, brain, eye, proventriculus, stomach, heart, kidney, liver, pancreas, muscle, and feces samples. Likewise, a clinical symptom of proventriculus dilation causing thinning of the gastrointestinal wall with undigested seeds was observed in Figure 5E of a PaBV-positive bird after suffering from PDD. The proventriculus measured about 6 cm in length over one-third to the overall length of the bird (about 18 cm) and had a 0.915 proventricular diameter-to-dorsoventral keel height ratio, exceeding the normal range for the proventricular diameter-to-dorsoventral keel height score. The diverse clinical manifestations observed in PaBV-positive psittacine birds, ranging from severe meningoencephalitis to gastrointestinal complications, underscore the multifaceted nature of the disease and the need for comprehensive veterinary care and management strategies.

For further analysis, the three brain samples (Bird cases 2208023, 2301024, and 2302010) were amplified for their X/P gene with 682 bp in length. Prior to X/P gene amplifications, the brain samples were tested for PaBV through RT-qPCR with C_T_ values of 18.59, 12.49, and 5.15 (Table 3 and Figure 5D). The organs were collected several days after the decease of the psittacine birds affecting viral RNA load and C_T_ values in different organs. The brain has consistently low C_T_ values reflecting high viral load and a much more suitable source for PCR amplification of the X/P gene. Therefore, it was selected for X/P gene amplification.

NCBI BLASTn search provided the homology analysis, which revealed that two of the Taiwan strains (NPUSTIAVT-PaBV/Brain-2208023 and NPUSTIAVT-PaBV/Brain-2302010) were PaBV-4, with both originating from Changzhi, Pingtung, and isolated from *Diopsittaca nobilis* (red-shouldered macaw), and one (NPUSTIAVT-PaBV/Brain-2301024) PaBV-2 was isolated from *Nymphicus hollandicus* (cockatiel) in Kangshan, Kaohsiung. The sequences were submitted and deposited in GenBank with accession numbers PP529446-PP529448.

The Taiwan strain PaBV-2 X/P gene (PP529446.1: NPUSTIAVT-PaBV/Brain-2301024) has 99.41% sequence similarity with the Germany strain PaBV-2 X/P gene (KU748803.1: L55595) from a *Psittacus erithacus* (African grey parrot). The two Taiwan strain PaBV-4 X/P genes, NPUSTIAVT-PaBV/Brain-2208023 (PP529447.1) and NPUSTIAVT-PaBV/Brain-2302010 (PP529448.1), have 95.89% sequence similarity with each other, with 85.51% and 84.77% sequence similarity with the Taiwan strain PaBV-2 X/P gene. The Taiwan strain PaBV-4 X/P gene, NPUSTIAVT-PaBV/Brain-2208023 (PP529447.1), has 99.71% sequence similarity with the Japan strain (LC486412.1: AR18A) isolated from the brain of an *Ara ararauna* (blue-and-yellow macaw), Germany strains (MK291400.1: L62637) from a *Psittacula cyanocephala* (plum-headed parakeet), (MK291396.1: DR-15) *Cacatua galerita* (sulfur-crested cockatoo), (KU748815.1: TiHo-154) *Ara glaucogularis* (blue-throated macaw), (KU748814.1: TiHo-40) *Ara chloroptera* (red-and-green macaw), Spain strain (MK291399.1: G-18720) from a *Psittacula alexandri* (red-breasted parakeet), and South Korea strains (MZ310179.1: CBNU_PaBV_04 and MZ310182.1: CBNU_PaBV_07) from an *Amazona ochrocephala* (yellow-crowned amazon). Lastly, the Taiwan strain PaBV-4 X/P gene, NPUSTIAVT-PaBV/Brain-2302010 (PP529448.1), has a 99.85% sequence similarity with Germany strain (KU748811.1: L63115) from an *Ara macao* (scarlet macaw).

**Figure 6:**
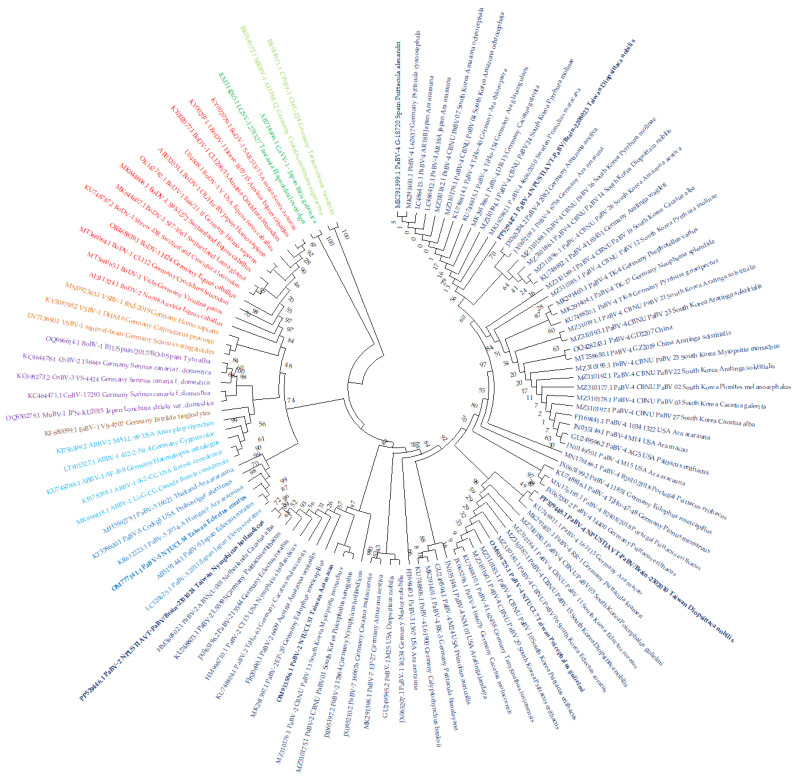
Maximum likelihood through GTR + I model at 1000 bootstrap replicates—phylogenetic tree of X/P gene sequence (682 base pairs in length). Each *Orthobornavirus* species has a different font color *Orthobornavirus alphapsittaciforme* (dark blue), *Orthobornavirus betapsittaciforme* (blue), *Orthobornavirus serini* (purple), *Orthobornavirus estrildidae* (brown), *Orthobornavirus avisaquaticae* (light blue), *Orthobornavirus bornaense* (red), *Orthobornavirus sciuri* (orange), *Orthobornavirus caenophidiae* (light green), and *Orthobornavirus elapsoideae* (dark green). The Taiwan strain X/P genes are highlighted in bold.

A phylogenetic tree was applied, using our sequenced X/P gene and all the available X/P gene sequences (682 nucleotides) of *Orthobornavirus alphapsittaciformes*, *Orthobornavirus betapsittaciformes*, and other genotypes of *Orthobornavirus*, with one representative per species isolates in each country. The analysis conclusively shows that our X/P gene sequence, Taiwan strain NPUSTIAVT-PaBV/Brain-2301024, belongs to the PaBV-2 genotype, and Taiwan strains NPUSTIAVT-PaBV/Brain-2302010 and NPUSTIAVT-PaBV/Brain-2208023 belong to the PaBV-4 genotype (Figure 6).

## 4. Discussion

Since the first report of avian bornaviruses in 2008, there has been an increase in the number of countries reporting the presence of PaBV, a novel infectious viral agent causing proventricular dilatation disease affecting psittacine birds [1,11,14,16,17,18,19,21,35,36,37,38,39,40,41]. In Taiwan, the presence of PaBV has been known through the submitted sequences of the matrix protein (M) gene (MK736729.1-MK736782.1), nucleoprotein (N) gene (MK770085.1-MK770118.1), and PaBV-2 (OM933586.1), PaBV-4 (OM939725.1), and PaBV-5 (OM777141.1) complete genome in GenBank [42]. To further elucidate the PaBV presence in Taiwan, we screened feces samples from 124 psittacine birds collected for a year (June 2022 to May 2023), and demonstrated that 57 were PaBV-positive, with a prevalence of 45.97% and a low survival rate of 8.77%. Feces samples were collected freshly and tested for RT-qPCR immediately to secure the presence of PaBV in the feces and reduce false negative results. The majority of the tested feces were collected in a local animal hospital where the psittacine owners brought them for either regular health evaluation or veterinary care concerns due to diarrhea or loss of appetite. This may influence the prevalence rate determined in this study, which may require further surveillance of parrot bornavirus in Taiwan.

In the detection of PaBV through RT-qPCR assay, C_T_ < 21 is strongly positive [30]; however, viral RNA present in the feces samples were observed to have a higher C_T_, carried out with the impurities in feces samples [11]. In the RT-qPCR detection, C_T_s higher than 21 were also observed as a possible outcome [20,30]. Gel electrophoresis of the RT-qPCR product may be suitable in securing and further confirming detection results to reduce false-negative or -positive results. Tissue samples such as the proventriculus, kidney, colon, cerebrum, and cerebellum have the most viral RNA, much more suitable for RT-qPCR detection [20]. However, viral RNA extraction and PCR detection should be performed promptly to minimize false results due to the unstable nature of the virus as single-strand negative-sense RNA. Delays can lead to degradation of the RNA, potentially yielding inaccurate results. Due to the sampling limitations, it is crucial to conduct further surveillance studies of PaBV in Taiwan. This would help in understanding the transmission dynamics and potential impact of PaBV on parrot populations, especially in Taiwan. Enhanced surveillance can provide valuable insights for disease management and prevention strategies, safeguarding both companion and wildlife psittacine birds.

Young psittacine birds are the most vulnerable to PaBV infection or PDD [43,44]; however, they may have no clinical signs but are the probable source of seroconversion and PaBV-RNA shedding [45]. In most cases, psittacine birds are housed together with other birds. Viral transmission sources to housed birds may come from newly introduced young bird/s in the enclosure, a potential carrier of PaBV. Then, PaBV shedding in the enclosure infects adult birds and possibly introduces the PaBV to the brood or clutch [44,46]. Adult psittacine birds appeared to have more clinical symptoms [43] and sex appeared to have no significant influence on PaBV infection. The age and sex of psittacine birds are often unknown to owners since most were adopted without any preexisting information. Moreover, among the tested PaBV-positive psittacine birds in Taiwan, it was more common in *Psittacus erithacus* or the African grey parrots (19%). Similar PaBV host species cases were reported in the United States, Japan, South Korea, China, Portugal, Thailand, Germany, Canada, Austria, Hungary, Switzerland, and Israel [10,12,14,25,26,27,28].

PDD affects the overall digestive health of the psittacine bird such as body weight, crop emptying, and seed digestion, alongside neurological problems such as severe meningoencephalitis [27,43,47]. Proventriculus dilation displayed thinning of the gastrointestinal wall and a build-up of undigested seeds that may cause pain and discomfort to psittacine birds, leading to a loss of appetite and further causing malnutrition, weight loss, depression, loss of balance, and tremors. Through radiograph scans, the proventricular diameter-to-dorsoventral keel height is determined, from 0.681 to 0.981, indicating the poor survival of the psittacine birds. The highly enlarged proventriculus and thinning wall are clinical signs of neurogenic atrophy. Histopathology of the cerebellum in deceased psittacine birds due to PaBV infection elucidates severe diffuse meningoencephalitis due to accumulated lymphocytes. The PaBV infection could cause PDD due to gastrointestinal crisis; however, neurological damage was more likely to appear in PaBV infection. Recently, it has become more appropriate to address this neurological crisis as avian ganglioneuritis (AGN) [48,49]. Probable mechanisms of PaBV leading to PDD/AGN were attributed to CD8+ T-cells, causing injury to neurons and ganglia recruiting CD4 T-cells, facilitating the antibody-mediated phagocytosis of axons [48]. This leads to persistent damage in the nervous system, suggesting an autoimmune response to gangliosides [48]. Histopathology of the heart showed a dilated cardiomyopathy or myocardium. This highlights the etiological implications of PaBV in neurological disorders other than PDD [38].

The spring season (68%) has the highest cases of PaBV infection while the summer season (28%) has the least, which might suggest that PaBV has a cycle transmission with regard to the season. Low PaBV infection during the summer season could be due to dry air, while the fall season could potentially allow the spread of PaBV in the environment, most probably due to dry feces particles in the air or fecal–oronasal route [11], viral trace contamination of waters, and residues of PaBV that are already present in the surroundings [1]. An increase in moisture or humidity during the winter season could contribute to viral persistence, spread, and incubation leading to contamination in feeds or drinking water, resulting in high cases of PaBV in the spring season and permitting a continuous incubation life cycle of PaBV [1]. The presence of PaBV in the environment and horizontal transmission to companion psittacine birds is still a challenge to understand [8]. Potential viral reservoirs such as wild free-ranging psittacine birds or other potential host carriers have not been fully illustrated, similar to PaBV host species which are most prevalent, and surveillance reports were carried out in captive, breeding, or companion psittacine birds [1,2,4,5,6,7,8,9,10,11,12,13,14,15,16,17,18,19,20,21,22,23,24,25,26,27,28,29,39,42,44,50,51]. In Taiwan, all psittacine birds were introduced, mostly kept as captive, companion, or breeding birds. Certainly, preventive measures targeting both horizontal and vertical transmission are vital for successfully managing PaBV infection cases [8,17,44,46,52,53], particularly in Taiwan.

Furthermore, our X/P gene sequences confirmed the presence of PaBV-2 and PaBV-4 genotypes in Taiwan strains. The two identified PaBV-4 X/P genes have 95.89% similarity from each other. Among our sequenced X/P genes, Germany strains were found to be consistently similar to our sequences. Taiwan strain NTUCL7 PaBV-4 has 95.89% sequence similarity with NPUSTIAVT-PaBV/Brain-2208023 and 99.7% sequence similarity with NPUSTIAVT-PaBV/Brain-2302010, while Taiwan strain NTUCL51 PaBV-2 has 97.80% sequence similarity with NPUSTIAVT-PaBV/Brain-2301024. Recently, phylogeographic analysis revealed that the South American ancestor could be the origin of PaBV-1, -2, and -8 genotypes [42]. This highlights the importance of further surveillance of *Orthobornavirus* for captive *Psittaciformes,* similar to Taiwan.

Moreover, avian bornavirus, specifically parrot bornavirus, causes severe neurological and digestive disorders or death in infected birds [14,16,36,54], a threat to psittacine birds of Taiwan. Additionally, it could disrupt local ecosystems affecting unique and vulnerable bird species and have economic implications for industries reliant on bird populations, such as agriculture, bird breeding farms, and tourism.

The findings of this study highlight the importance of the ongoing monitoring of Parrot bornavirus in Taiwan’s psittacine populations to monitor new strains or potential recombinants, as demonstrated in a recent report [2].

## Figures and Tables

**Table 1:** List of PaBV-positive rates in different psittacine species.

Psittacine Species of PaBV-Positive	Count	Rate
*Psittacus erithacus*	11	19%
*Nymphicus hollandicus* and *Aratinga solstitialis*	8	14%
*Diopsittaca nobilis*, *Myiopsitta monachus*, and *Amazona ochrocephala*	4	7%
*Cacatua ophthalmica*, *Eclectus roratus*, *Ara severus*, *Pionites leucogaster*, *Amazona aestiva*, *Agapornis* sp., and *Psittacula krameri*	2	4%
*Poicephalus gulielmi*, *Pyrrhura molinae*, *Melopsittacus undulatus*, and *Cacatua moluccensis*	1	2%

**Table 2:** Summary of information for PaBV-positive cases. C_T_ values were obtained from feces samples.

Case Number	Bird Species	Location	Age	Sex	C_T_	Symptoms
2206003	*Myiopsitta monachus*	Renwu, Kaohsiung	1 y	-	27.47	a b c d e
2208002+	*Amazona ochrocephala*	Changjhih, Pingtung	3.5 y	Female	27.83	a b d e
2208008+	*Amazona ochrocephala*	Changjhih, Pingtung	3.5 y	Male	24.54	a b d e
2208023	*Diopsittaca nobilis*	Changjhih, Pingtung	2 y	-	23.99	a b c d e
22080061+	*Cacatua ophthalmic*	Yongkang, Tainan	1 y	-	28.43	a b d e
22080071	*Cacatua ophthalmica*	Yongkang, Tainan	1 y	-	27.30	a b d e h
22080091	*Diopsittaca nobilis*	Changjhih, Pingtung	2 y	-	27.26	a b c d e h
2208013+	*Psittacus erithacus*	Renwu, Kaohsiung	4 y	Male	27.38	a b c d
2209009	*Pionites leucogaster*	Taibao, Chiayi	-	-	27.54	a b d e
2210003	*Nymphicus hollandicus*	Yongkang, Tainan	3 y	Male	26.81	a b c d e
2210004	*Eclectus roratus*	Linyuan, Kaohsiung	-	Male	27.89	a b d e
22110011	*Amazona ochrocephala*	Changjhih, Pingtung	3.5 y	Male	26.43	a b d e
22110012	*Ara severus*	Changjhih, Pingtung	1.5 y	Male	27.48	a b d e
22110013	*Ara severus*	Changjhih, Pingtung	1.5 y	Female	29.00	a b d e
22110014	*Amazona ochrocephala*	Changjhih, Pingtung	3.5 y	Female	27.40	a b d e
2301024	*Nymphicus hollandicus*	Gangshan, Kaohsiung	4 y	Female	26.36	a b c d e h
2212037	*Poicephalus gulielmi*	Alian, Kaohsiung	-	-	29.34	a b c d e
2212064	*Pyrrhura molinae*	Dapi, Yunlin	-	-	30.29	a b c d e
2302010	*Diopsittaca nobilis*	Pingtung, Pingtung	-	Female	23.47	a b c d e
2302013+	*Psittacus erithacus*	Yongkang, Tainan	5 y	Male	27.88	a b d e f g
2302017	*Psittacus erithacus*	Yongkang, Tainan	3 y	Female	27.66	a b c d e h
2302025	*Nymphicus hollandicus*	Gangshan, Kaohsiung	1 y	Female	28.25	a b c d e
2302026	*Nymphicus hollandicus*	Gangshan, Kaohsiung	1 y	Male	28.48	a b c d e h
2303007	*Psittacus erithacus*	Yongkang, Tainan	3 y	Male	27.68	a b c d e
2303008	*Melopsittacus undulatus*	Chiayi, Chiayi	-	Female	28.31	a b c d e
2303013	*Agapornis* sp.	Gangshan, Kaohsiung	-	Male	29.30	a b d e g h
2303014	*Agapornis* sp.	Gangshan, Kaohsiung	-	Male	28.97	a b d e
2303017	*Eclectus roratus*	Linyuan, Kaohsiung	-	Male	24.34	a b c d e
2303019	*Psittacus erithacus*	Fengshan, Kaohsiung	3 y	Female	27.66	a b c d e h
2303023	*Nymphicus hollandicus*	Nanzi, Kaohsiung	-	Male	29.80	a b c d e
2303030	*Psittacula krameri*	Taoyuan, Taoyuan	-	Male	25.13	a b d e
2304002	*Nymphicus hollandicus*	Fengshan, Kaohsiung	-	-	22.80	a b c d e
2304003	*Psittacus erithacus*	Yongkang, Tainan	4 y	Male	31.32	a b d e
2304004011	*Myiopsitta monachus*	Xiaogang, Kaohsiung	-	-	24.91	a b c d e
2304004021	*Myiopsitta monachus*	Xiaogang, Kaohsiung	-	-	19.93	a b c d e
2304005	*Myiopsitta monachus*	Wantan, Pingtung	-	-	26.25	a b d e
2304006	*Aratinga solstitialis*	Wantan, Pingtung	-	-	23.51	a b d e
2304007	*Diopsittaca nobilis*	Taoyuan, Taoyuan	-	Male	28.68	a b c d e
2304008	*Psittacus erithacus*	Yanchao, Kaohsiung	-	Female	27.76	a b c d e g h
2304015	*Psittacula krameri*	Yongkang, Tainan	-	-	28.30	a b d e
2304018	*Pionites leucogaster*	Yongkang, Tainan	-	-	28.93	a b d e
2304019	*Aratinga solstitialis*	Yongkang, Tainan	-	-	29.78	a b d e
2304020	*Amazona aestiva*	Yongkang, Tainan	-	-	30.05	a b d e
2304021	*Aratinga solstitialis*	Yongkang, Tainan	-	-	30.15	a b d e
2304022	*Amazona aestiva*	Yongkang, Tainan	-	-	28.79	a b c d e
2304025	*Nymphicus hollandicus*	Wujih, Taichung	-	-	29.32	a b c d e
2304026	*Nymphicus hollandicus*	Qianzhen, Kaohsiung	-	-	29.49	a b c d e
2304028	*Aratinga solstitialis*	Chiayi, Chiayi	-	-	28.46	a b d e g h
2304029	*Aratinga solstitialis*	Chiayi, Chiayi	-	-	28.87	a b d e
2304030	*Aratinga solstitialis*	Chiayi, Chiayi	3 y	Male	28.94	a b d e
2304031	*Aratinga solstitialis*	Chiayi, Chiayi	-	-	31.15	a b d e
2304032	*Aratinga solstitialis*	Chiayi, Chiayi	-	-	30.16	a b d e
23050071	*Psittacus erithacus*	Wantan, Pingtung	-	Male	30.40	a b c d e g h
23050081	*Psittacus erithacus*	Wantan, Pingtung	-	Male	31.81	a b c d e g h
2305009	*Psittacus erithacus*	Wantan, Pingtung	-	Male	27.25	a b c d e g h
2305025	*Psittacus erithacus*	Dapi, Yunlin	-	Female	28.92	a b c d e g h
2305026	*Cacatua moluccensis*	Yanshui, Tainan	-	-	28.57	a b c d e g h

(-) The information is unknown. Symptoms: weight loss (a), crop stasis (b), regurgitation (c), maldigestion (d), depression (e), seizure (f), ataxia (g), and tremors (h). (+) The bird successfully recovered from the infection.

**Table 3:** Summary of the C_T_ values in different organs for PaBV detection.

Case No.	Genotype	Brain	Lung	Pancreas	Stomach	Intestine	Liver	Eye	Kidney	Heart	Muscle	Proven.
2208023	PaBV-4	18.59	23.3	23.38	23.98	24.29	27.98	-	-	-	-	-
2301024	PaBV-2	12.94	24.35	25.3	26.43	25.76	29.17	18.52	21.6	24.15	29.24	20.22
2302010	PaBV-4	5.15	6.09	7.88	4.27	7.57	7.75	4.94	4.4	25.97	-	-

(-) The information is unknown as the organ is not tested or the organ is unavailable.

## Data Availability

All data generated and analyzed in this study are included in this article. PaBV X/P gene nucleotide sequences were deposited in GenBank with accession numbers PP529446-PP529448.
